# The implication of aberrant NRF2 activation in management of female cancers

**DOI:** 10.3389/fonc.2025.1579135

**Published:** 2025-11-17

**Authors:** Mankgopo Kgatle, Saidon Mbambara, Olalekan Fadebi, Joseph Kabunda, Chimbabantu Kaoma, Thobeka Dlangalala, Siphesihle Nxele, Ndimo Modipane, Thato Serite, Kgomotso Mokoala, Tivani Mashamba-Thompson, Mike Sathekge

**Affiliations:** 1 Nuclear Medicine Research Infrastructure, Department of Basic and Translational Research, Pretoria, South Africa; 2 Department of Nuclear Medicine, University of Pretoria, Pretoria, South Africa; 3 Department of Medicine, University of Cape Town and Groote Schuur Hospital, Observatory, Cape Town, South Africa; 4 Department of Biomedical Sciences, Tropical Diseases Research Centre, Ndola, Zambia; 5 School of Health System and Public Health, Faculty of Health Sciences, University of Pretoria, Pretoria, South Africa

**Keywords:** NRF2 activation, oxidative stress, tumor progression, therapy resistance, gynecological cancers, targeted inhibitors, PET imaging, personalized treatment

## Abstract

The overactivation of NRF2 (Nuclear factor erythroid 2-related factor 2) in female malignancies is an emerging field of study with significant implications for treatment efficacy. NRF2 plays a pivotal role in managing inflammation-induced oxidative stress, which is crucial components of the tumor microenvironment. Acting as a transcription factor and basic leucine zipper protein, it regulates the expression of various antioxidant genes that safeguard cells from oxidative stress and damage. While NRF2 activation is beneficial for the survival of normal cells, its overactivation in cancer cells can enhance tumor cell survival, proliferation, and resistance to treatments. Importantly, NRF2 has a dual context-dependent role, functioning as a tumor suppressor when transiently activated in normal cells to prevent carcinogenesis, but as an oncogene when persistently activated in established tumors. Understanding NRF2’s transcriptional alterations and developing targeted therapies could improve cancer management, prognosis and treatment outcomes, making it a promising target for precision oncology. This review aims to provide a comprehensive overview of NRF2 activation in female malignancies, including cervical, endometrial, ovarian, vaginal, vulvar and, breast cancers, and its association with chemoresistance, highlighting challenges and opportunities for developing more effective cancer treatments.

## Introduction

Female cancers including breast and gynecological cancers significantly impact women’s health (1, 2). Gynecological cancers include cervical, endometrial (uterine), ovarian, vaginal, and vulvar cancers. Although not classified as a gynecological cancer, breast cancer also significantly impacts female reproductive health (3, 4). Both breast and gynecological cancers require early detection and specialized treatment to improve outcomes and survival rates. Treatment options vary depending on the type and stage of the cancer and may include surgery, radiation therapy, chemotherapy, and targeted therapies. However, treatment resistance remains a considerable hurdle.

These cancers can greatly affect reproductive health and overall well-being, presenting symptoms like abnormal bleeding, pelvic pain, and unusual discharge (1, 5). Late detection is common, particularly with ovarian cancer, which often manifests with nonspecific symptoms such as bloating and abdominal pain. These symptoms result from cancerous cells disrupting the normal functions of the female reproductive organs (6). The functioning of these organs relies heavily on the transcriptional regulation of various transcription factors that govern specific gene transcription necessary for cell growth, differentiation, and proper functioning (7). Any alterations in the activity or levels of these transcription factors can lead to disruptions in normal reproductive functions, potentially causing irregularities, infertility, infections, and diseases such as cancer (7). Improving early detection through regular screenings and increased symptom awareness is crucial for better treatment outcomes and patient prognosis.

Genetic alterations, such as mutations in *BRCA1* and *BRCA2* genes, play a critical role in the development, progression, and spread of breast and certain gynecological cancers. These genes are involved in DNA repair, and mutations can lead to heightened oxidative stress and genomic instability (8). Chronic inflammation contributes to cancer progression by fostering a microenvironment that activates multiple transcriptional regulation mechanisms. For example, inflammatory signaling pathways such as nuclear factor kappa B (NF-kB) and signal transducer and activator of transcription 3 (STAT3) regulate genes linked to cancer progression (9). Additionally, cytokines produced by inflammatory cells, including tumor necrosis factor-alpha (TNF-α), interleukin-6 (IL-6), and interleukin-1 beta (IL-1β), activate transcription factors that promote cell proliferation, survival, and angiogenesis. Additionally, epigenetic modifications, such as DNA methylation and histone changes, alter gene expression, and post-transcriptional processes affect mRNA stability and translation. These combined mechanisms significantly contribute to the onset, progression, and metastasis of female cancers (10–12).

**Figure 1:**
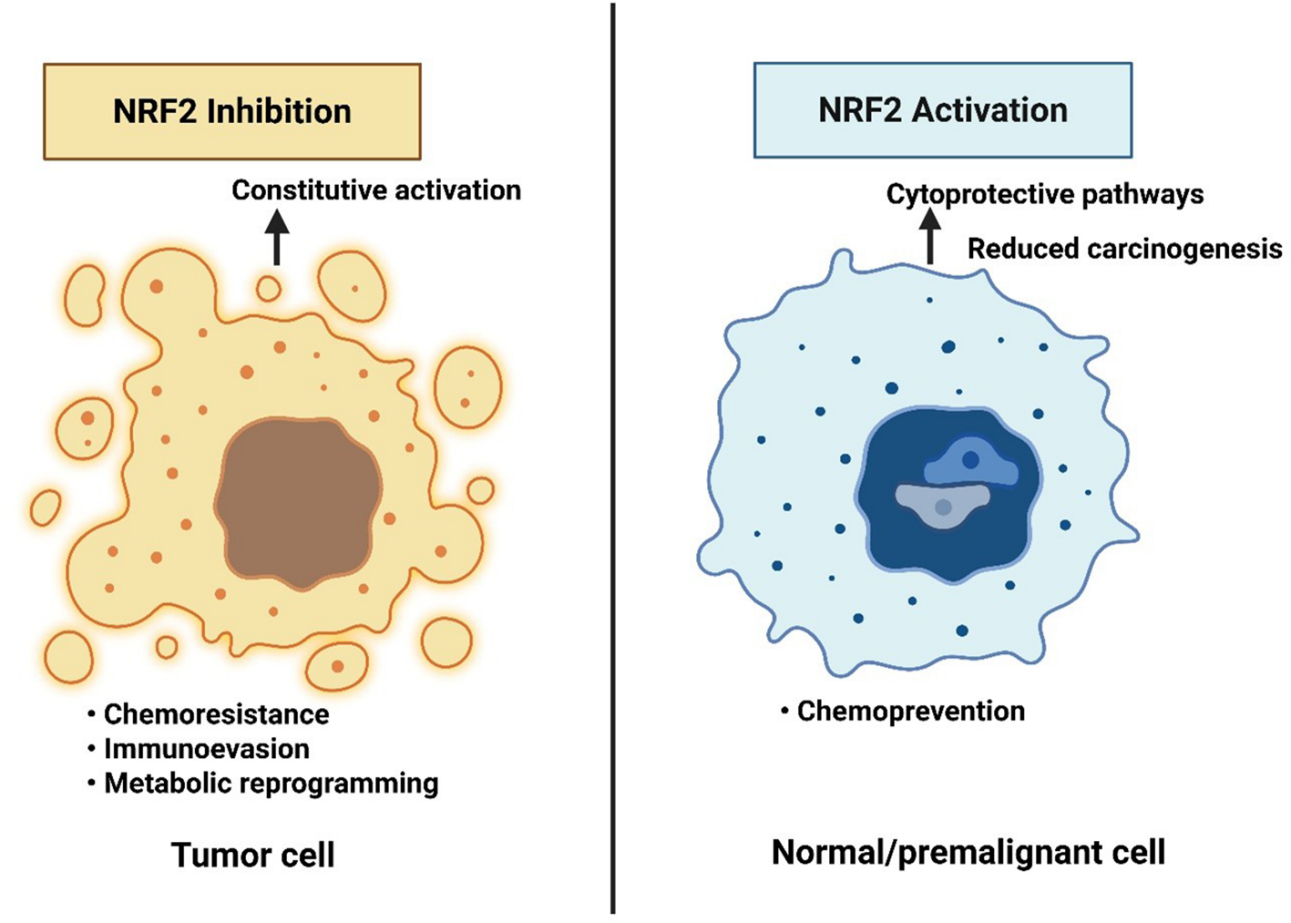
The NRF2 paradox in cancer therapy and prevention. Constitutive NRF2 activation in tumor cells promotes chemoresistance, immune evasion, and metabolic reprogramming, supporting malignant progression. In contrast, NRF2 activation in normal or premalignant cells enhances cytoprotective pathways and reduces carcinogenesis. This dual role highlights the therapeutic paradox: NRF2 inhibition may benefit established cancers, whereas NRF2 activation may serve as a chemopreventive strategy in high-risk but non-malignant contexts.

NRF2 exhibits a dual role in cancer biology. In normal or pre-malignant cells, transient NRF2 activation acts tumor-suppressive by reducing ROS-induced DNA damage and carcinogenesis (13). Conversely, in established tumors, persistent NRF2 hyperactivation, often through KEAP1 mutations or epigenetic dysregulation, becomes oncogenic, promoting proliferation, metabolic reprogramming, and chemoresistance (13). This context dependency underpins the paradox of NRF2 as both ‘guardian’ and ‘driver’ of cancer, requiring careful therapeutic consideration, as illustrated in **Figure 1**. NRF2 not just as a redox regulator but as a driver of multiple cancer hallmarks, reinforcing its relevance in female cancers like breast, ovarian, and endometrial malignancies (13).

Overactivation of NRF2 may be linked to female cancers, some of which may be aggressive and exhibit distinct molecular features (14, 15). Mutations in KEAP1, hypoxic environment, along with epigenetic and post-translational alterations, lead to persistent NRF2 activation, enhancing cancer cells’ antioxidant capabilities and metabolic flexibility (16). This provides a survival advantage by neutralizing ROS and detoxifying chemotherapeutic agents. While transient NRF2 activation protects against carcinogenesis, its persistent activation may lead to malignant progression, increased aggressiveness, therapy resistance, and poor prognosis (17–19). NRF2 upregulates antioxidant and detoxifying genes, counteracting oxidative stress from chronic inflammation, influencing inflammatory responses, and maintaining cancer stem cells, which contribute to therapy resistance and tumor relapse (20–23).

This review aims to provide an overview of NRF2 activation in female malignancies and its association with chemoresistance, highlighting challenges and opportunities for developing more effective cancer treatments.

## Activation of NRF2 and general implication in cancer

The NRF2 transcription factor plays a central role in regulating antioxidant and detoxification systems in response to oxidative and electrophilic stress (20–23). Reactive oxygen species (ROS), normally generated under homeostatic conditions, are essential signaling molecules that regulate proliferation, differentiation, and survival (19, 24). They also function as second messengers in extracellular signal transduction, coordinating communication within and between cells (19). However, excessive ROS and reactive nitrogen species (RNS) lead to oxidative stress, which fosters carcinogenesis by driving oncogene activation, inactivation of tumor-suppressor pathways, and mitochondrial dysfunction (19, 24).

The biological consequences of NRF2 activation are highly context-dependent. In normal physiology and early stages of carcinogenesis, NRF2 preserves redox balance, prevents ROS-induced mutagenesis, and thus acts as a tumor suppressor (22). By contrast, in established cancers, persistent NRF2 signaling is often hijacked by tumor cells to sustain anabolic metabolism, block apoptosis, and promote resistance to chemotherapy and radiotherapy (25). This duality underpins NRF2’s reputation as both a “protector” of normal tissue and an “enabler” of cancer progression (25).

**Figure 2:**
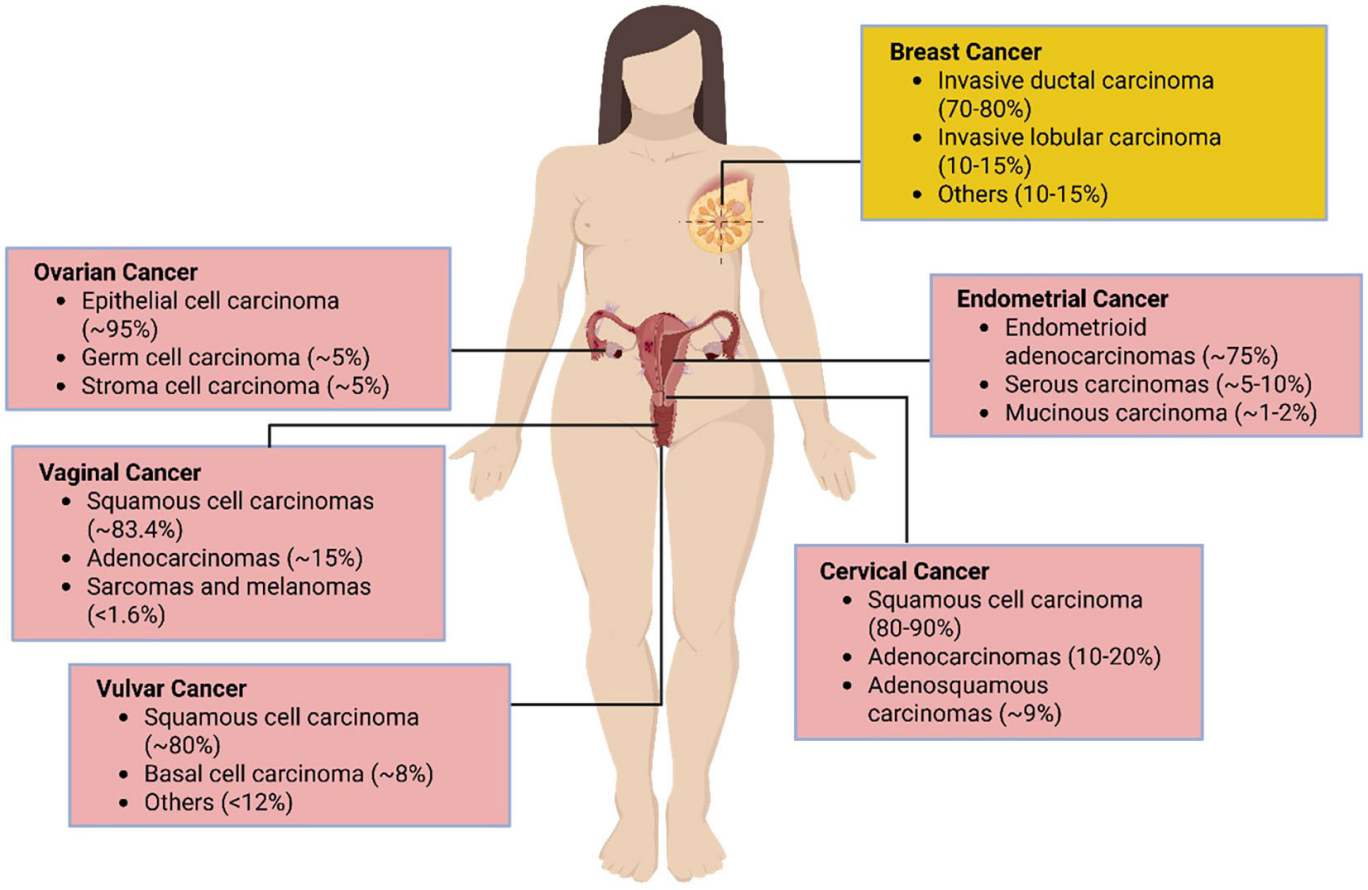
Activation of NRF2 through ROS leads to the dissociation of NRF2 from the NRF2/CUL3/KEAP1 complex, allowing the NRF2 to translocate into the nucleus. In the nucleus, the NRF2 binds to the ARE, forming an NRF2/ARE complex and thereby regulating the transcription of its target genes. In its inactivated form, NRF2 levels are continuously kept low through proteasomal degradation in the cytoplasm. NRF2 activation pathway, showing KEAP1-NRF2 regulation, downstream antioxidant targets (GCLC, NQO1, HMOX1, TXNRD1, MRPs, GST), and potential pharmacological intervention points relevant to female cancers.

At the molecular level, NRF2 controls the expression of genes that drive metabolic reprogramming and encode antioxidant and detoxifying enzymes. Under basal conditions, NRF2 is sequestered in the cytoplasm by its inhibitor KEAP1 (as illustrated in **Figure 2**), which forms a complex with the CUL3-based E3 ubiquitin ligase to target NRF2 for proteasomal degradation, thereby maintaining low NRF2 levels (26). Upon exposure to stressors such as ROS, modifications or mutations in KEAP1 disrupt this interaction, allowing NRF2 to escape degradation, accumulate in the nucleus, and dimerize with small MAF (sMAF) proteins (26). The NRF2-sMAF complex binds to antioxidant response elements (AREs) in target gene promoters, initiating transcriptional programs that enhance antioxidant defense, detoxification, and cellular repair.

Key NRF2 target genes include *glutamate-cysteine ligase catalytic subunit* (*GCLC*), *NAD(P)H quinone oxidoreductase 1* (*NQO1*), *heme oxygenase-1* (*HMOX1*), *UDP-glucuronosyltransferase* (*UGT*), *sulfiredoxin 1* (*SRXN1*), *thioredoxin reductase 1* (*TXNRD1*), *multidrug resistance-associated proteins* (*MRPs*), and *glutathione S-transferase* (*GST*) (27). Together, these effectors underscore NRF2’s central role in maintaining cellular redox homeostasis and influencing cancer progression.

## Key targets pathways for NRF2 activation

### Glutamate-cysteine ligase catalytic subunit

Glutathione (GSH) is a critical tripeptide antioxidant involved in protein synthesis, DNA repair, and detoxification via conjugation reactions (28, 29). Its biosynthesis begins with the rate-limiting step catalyzed by γ-glutamylcysteine ligase (GCL), composed of a catalytic (GCLC) and a modifier (GCLM) subunit (30). GCLC links glutamate to cysteine, while GCLM enhances this efficiency (31). Oxidative stress strongly regulates GCL activity, which is essential for sustaining cellular GSH levels and protecting mitochondria from toxicity (32–34). In turn, GSH scavenges reactive oxygen species (ROS) and other free radicals, maintaining redox balance (32, 35).

Cysteine availability is the major constraint in GSH synthesis. It enters cells primarily as cystine via the cystine/glutamate antiporter xCT (SLC7A11) and is then reduced to cysteine by intracellular GSH or the NADPH-dependent thioredoxin system (36). Overexpression of xCT/SLC7A11 has been reported in triple-negative breast cancer (TNBC), where its activity fuels cysteine supply for GSH production; conversely, xCT inhibition reduces proliferation, partly through GSH depletion (37).

Mechanistically, NRF2 transcriptionally regulates GCL by binding to AREs in the promoter regions of the GCLC and GCLM genes, thereby enhancing glutathione biosynthesis and enabling cells to sustain redox homeostasis under oxidative stress. In cancer, particularly gynecological malignancies, dysregulation of the KEAP1-NRF2 axis results in persistent NRF2 activation, which upregulates GCL expression and promotes chemoresistance (38, 39). While some cancers exhibit reduced GCL activity and low glutathione levels, gynecological cancers such as endometrial and ovarian subtypes often display elevated GCLC expression, conferring metabolic flexibility and resistance to therapy. For instance, ARID1A-deficient ovarian cancers demonstrate synthetic lethality upon GCLC depletion, underscoring its therapeutic potential (31, 40).

Beyond regulating GCL, NRF2 also induces SLC7A11 (xCT) and GPX4, strengthening cellular defenses against ferroptosis and allowing tumor cells to evade iron-dependent cell death (15). In TNBC, mesenchymal stem-like cells exploit cystine uptake via xCT/SLC7A11 to activate NRF2, establishing antioxidant programs that extend beyond GSH synthesis. This cystine/NRF2/OSGIN1 axis promotes stemness, EMT, and survival, with NRF2-induced genes such as OSGIN1, SRXN1, and AKR1B10 linked to poor prognosis, highlighting a potential therapeutic vulnerability in aggressive TNBC (41).

Abnormal GSH levels are also linked to increased GCL expression or activity in cancers such as liver, lung, breast, and malignant mesothelioma, correlating with metastasis, chemoresistance, and poor prognosis (27). High GCLC expression has been consistently observed in multiple cancers, supporting its utility as a biomarker and therapeutic target (42). Additionally, NRF2-GCL deregulation has been reported in endometriotic lesions, where it contributes to growth and fibrosis (27). CRISPR knockout studies further demonstrate that GCLC is essential for the survival of acute myeloid leukemia (AML) cells but less critical for normal hematopoietic cells, highlighting its selective therapeutic potential (43, 44). Despite these insights, the expression and function of GCLC and GCLM in female cancers remain insufficiently defined, underscoring the need for further research.

### NAD(P)H quinone oxidoreductase 1

*NQO1* is one of the most robustly induced NRF2 targets. It is also a versatile, FAD-dependent flavoprotein that promotes 2-electron reductions of quinones and other substrates, reducing oxidative stress by minimizing ROS generation and thiol depletion through p53 tumor suppressor stabilization (45). Its expression is tightly linked to NRF2 activity and is often used as a surrogate marker of NRF2 pathway activation in cancer studies. NQO1 is highly inducible and regulated by the Keap1/Nrf2/ARE pathway (46). Its antioxidant function in combating oxidative stress is crucial, as evidenced by studies showing that NQO1 induction decreases susceptibility to oxidative stress, while its depletion increases it. NQO1 also stabilizes tumor suppressor *p53* by preventing its degradation (46). Human polymorphisms suppressing NQO1 activity are linked to increased disease predisposition (47). NQO1 thus acts as a cytoprotective enzyme, providing antioxidant protection and regulating the degradation of specific proteins.

NQO1-deficient mice show lower p53 induction, reduced apoptosis, increased tumor susceptibility, and impaired NF-kB function. It has been demonstrated that the missense variant NQO1, found in 4-20% of the human population, results in no detectable NQO1 activity due to rapid degradation of the NQO1 P187S protein. Despite ongoing debates about the role of NQO1 in cancer, it has been found to be highly expressed in various cancers, including breast cancer, melanoma, lung cancer, cholangiocarcinoma, and pancreatic cancer (48–52). NQO1 homozygous individuals have a higher risk of benzene-induced hematotoxicity, acute nonlymphocytic leukemia, and other cancers, particularly leukemia. The variant also correlates with an increased risk of relapse or death in children treated for acute lymphoblastic leukemia and has been linked to increased breast cancer risk under specific conditions (53). Previous immunohistochemistry study has revealed that NQO1 plays a crucial role in the progression of gastric cancer, distinguishing it from non-cancer patients and healthy controls (49). Additionally, bioinformatics analysis of TCGA data also revealed significantly higher GCLC and GCLM expression in gastric adenocarcinoma tissues compared to normal tissues. Immunohistochemistry confirmed these findings, with 77% and 80% of gastric adenocarcinoma tissues showing positive staining for GCLC and GCLM, respectively, versus only 9% and 11% in adjacent normal tissues (54). The study concluded that dysregulated expression of GCLC and GCLM could promote tumorigenesis, presenting potential targets for valuable diagnostic or prognostic biomarker and therapeutic target.

Recent findings further underscore the oncogenic versatility of NQO1, particularly in gynecological malignancies. Elevated NQO1 expression in ovarian cancer has been shown to confer resistance to quinone-based chemotherapeutics and promote tumor survival, highlighting the dual role of NRF2 in cytoprotection and oncogenic adaptation (55). A study investigating ferroptosis, a regulated form of cell death driven by iron and lipid peroxidation, identified NQO1 as a key molecular target (56). Daphnetin (Daph), a natural compound, was found to directly bind and inhibit NQO1, thereby increasing oxidative stress and inducing ferroptosis in ovarian cancer cells. This inhibition significantly reduced cell proliferation and migration *in vitro* and *in vivo* (56). Notably, overexpression of NQO1 reversed these effects, confirming its protective role in ovarian cancer cell survival. These findings position NQO1 not only as a modulator of ferroptosis but also as a promising therapeutic target, especially in combination with agents like cisplatin.

### Heme oxygenase-1

HMOX1 is a 32kDa key metabolic enzyme involved in the mammalian stress response (57). It has two major isoforms, HO-1 and HO-2, which degrade heme to carbon monoxide (CO), ferrous iron (Fe²^+^), and biliverdin (BV) (58, 59). HO-1 is inducible by stress factors like cadmium, hydrogen peroxide, hypoxia, and inflammation, while HO-2 is constitutively expressed (60, 61). Both isoforms localize to the endoplasmic reticulum but can redistribute under stress. Despite lacking heme groups, it plays essential roles, including catalyzing heme degradation with NADPH-Cytochrome P450 Reductase support. During injury healing, this process shifts heme to biliverdin and finally to bilirubin, facilitated by intramolecular hydroxylation of the heme molecule (62). HO-1 is found in many human tissues, especially the spleen, liver, and bone marrow, and plays a crucial role in iron homeostasis and protection against oxidative stress and inflammation (63). Notably, HMOX1 deficiency can be fatal, and this is evidenced by HO-1-deficient mice studies, which showed increased vulnerability to anemia, iron deposition, and oxidative stress-induced liver damage (64). Additionally, HO-1 influences bone metabolism and immune defense mechanisms, underlining its significance in various physiological processes.

The NRF2 also targets HMOX1 and breaks it down into beneficial products, and protects against various pathologies. NRF2 overactivation by early growth response 1 (EGR1) leads to increased HMOX1 transcription in breast cancer, providing a survival advantage to the cancer cells and promoting resistance to therapy (65). HMOX1 expression is notably higher in colon cancer cells compared to normal colonic epithelial cells across various hemin concentrations (66). However, in colorectal cancer tissues, HMOX1 expression is significantly lower than in adjacent non-neoplastic tissues (67). This suggests that while HMOX1 may provide cytoprotection within a certain range, excessive levels might contribute to tumor progression. Essentially, there’s a beneficial threshold where HMOX1 helps protect cells, but beyond this limit, it may act as an oncogene, promoting cancer development.

### UDP-glucuronosyltransferase

Another NRF-2 targets, UGT, SRXN1 and TXNRD1, support the reduction of peroxiredoxins, aiding in the detoxification of harmful peroxides. UGTs are a superfamily of enzymes found in animals, plants, fungi, and bacteria. They add sugars from UDP-sugar donors to various lipophilic molecules, aiding in the detoxification and elimination of harmful substances (68). In mammals, the superfamily has four families: UGT1, UGT2, UGT3, and UGT8. UGT1 and UGT2 families, particularly, are involved in pharmacology and toxicology, conjugating glucuronic acid to make molecules water-soluble for excretion. Genetic variations in UGTs affect drug metabolism and disease susceptibility. UGT3 and UGT8, using different sugars, may primarily conjugate endogenous chemicals, impacting cellular functions and potentially influencing cancer and degenerative diseases (68).

In cancer, UGTs have a dual role. On a positive note, they safeguard normal cells by eliminating harmful substances, such as xenobiotics and endogenous compounds, through glucuronic acid conjugation (14). However, the overactivation of NRF2 upregulates these drug-metabolizing enzymes, leading to enhanced drug metabolism and clearance in cancers like lung, colorectal, breast, and prostate cancer. This rapid elimination of chemotherapy drugs reduces their effectiveness, enabling cancer cells to survive and proliferate (14). Consequently, higher doses of drugs are often required, which can increase toxicity and side effects for the patient.

### Sulfiredoxin 1

A sulfiredoxin-1 is an antioxidant enzyme that catalyses the reduction of over-oxidized peroxiredoxins, restoring their active form (69). This reaction involves converting peroxiredoxin-(S-hydroxy-S-oxocysteine) to peroxiredoxin-(S-hydroxycysteine) using ATP and a thiol, producing ADP, phosphate, and a disulfide as by-products. Sulfiredoxins belongs to the thiol-based antioxidant system and can lower ROS under oxidative stress (70). They are also known by names such as Srx1 and peroxiredoxin-(S-hydroxy-S-oxocysteine) reductase. SRXN1 is essential for regulating various physiological processes like cell apoptosis, proliferation, invasion, and maintaining redox balance (71).

SRXN1 also plays a role in promoting carcinogenesis and protecting against ischemia/reperfusion injury in cardiac progenitor cells. When NRF2 is overactivated, it boosts the levels of SRXN1, which lowers ROS and helps cancer cells endure oxidative stress, thereby supporting their growth and survival (72). For instance, elevated levels of SRXN1 are associated with poor prognosis in several types of cancer, including non-small-cell lung cancer (NSCLC) and prostate cancer. Consequently, this leads to chemoresistance and radiotherapy resistance, thereby contributing to a more aggressive cancer phenotype with poorer patient prognosis. Another study has also identified SRXN1 as pro-tumorigenic in hepatocellular carcinoma (HCC) by influencing ROS signaling (73). Furthermore, SRXN1’s role in oxidoreductase activity suggested that its overexpression is critical in the tumorigenesis and progression of HCC (73).

### Thioredoxin reductase 1

*TXNRD1* gene encodes a pyridine nucleotide oxidoreductase that functions in selenium metabolism and oxidative stress protection by reducing thioredoxins and other substrates (74). The enzyme operates as a homodimer using FAD as a cofactor, with each subunit containing a selenocysteine (Sec) residue crucial for its catalytic activity. This residue is encoded by the UGA codon, typically a stop signal but recognized as Sec due to a common stem-loop structure in the 3’ UTR called the SECIS element (75). A thioredoxin-mediated reducing environment is essential for the effective DNA binding of redox-sensitive transcription factors like p53 and NF-κB. By binding ROS, thioredoxin protects against oxidative stress (76). Additionally, thioredoxin activates ribonucleotide reductase, inhibits apoptosis signal regulating kinase 1, and induces HIF-1 and vascular endothelial growth factor (VEGF), contributing to cancer traits such as increased proliferation, inhibited apoptosis, and angiogenesis.

TXNRD1 is a direct transcriptional target of NRF2, regulated through AREs located within its promoter region (77). Upon NRF2 activation, typically triggered by oxidative stress or KEAP1 disruption, the transcription factor translocates to the nucleus and induces TXNRD1 expression (26). This upregulation supports thioredoxin-mediated redox balance, selenium metabolism, and cellular protection against ROS-induced damage (41).

The cytoprotective function of TXNRD1 is particularly significant in cancer, where its overexpression is frequently observed across multiple malignancies, including lung, breast, and pancreatic cancers (78). In lung cancer, elevated TXNRD1 levels contribute to resistance against therapies such as cisplatin by maintaining low intracellular ROS levels, thereby shielding cancer cells from oxidative damage (78). In breast cancer, high TXNRD1 expression correlates with poor prognosis and increased metastatic potential, underscoring its role in tumor progression (79). Similarly, in pancreatic cancer, the thioredoxin system, of which TXNRD1 is a key component, enables cancer cells to evade oxidative stress-induced cell death (80). In non-small cell lung cancer (NSCLC), TXNRD1 overexpression, driven by NRF2 signaling, has been associated with tumor recurrence, adverse clinical outcomes, and chemoresistance, ultimately enhancing cancer cell survival (81).

Beyond its role as a downstream effector, TXNRD1 also engages in a dynamic feedback loop within the NRF2 regulatory network. When TXNRD1 levels are reduced, whether due to treatment-induced oxidative stress or post-translational modifications, compensatory NRF2 responses are triggered. These include increased nuclear accumulation of NRF2 and transcriptional activation of other ARE-regulated genes such as NQO1 and AOX1, aimed at restoring redox equilibrium (78). Consequently, TXNRD1 expression reflects both direct NRF2-mediated transcriptional control and adaptive feedback mechanisms responding to redox imbalance.

This dual regulatory role highlights the complexity of NRF2 signaling in cancer biology. Notably, the expression of NRF2 target genes may not consistently mirror pathway activation, particularly under therapeutic pressure or within heterogeneous tumor microenvironments. Such context-dependent modulation has important implications for interpreting TXNRD1 as a biomarker or therapeutic target in NRF2-driven malignancies.

### Multidrug resistance-associated proteins

NRF2 upregulates MRPs, a family of transporters that move various molecular substrates, including drugs, across cell membranes, affecting their pharmacokinetics and toxicity (22, 82). MRPs are part of the ATP-binding cassette (ABCC) superfamily and are differentially expressed in various organs, including the liver, kidney, intestine, and blood-brain barrier. There are nine human MRPs, which transport a diverse array of endogenous and exogenous compounds and their conjugates (83, 84). MRP1 has a high affinity for leukotriene C4, MRP2 extrudes endogenous organic anions such as bilirubin glucuronide and certain anticancer agents, and MRP3 transports glutathione and glucuronate conjugates, as well as monoanionic bile acids (83, 84). MRP4 and MRP5 mediate the transport of cyclic nucleotides and confer resistance to some antiviral and anticancer nucleotide analogues, while MRP6 is involved in transporting glutathione conjugates and the cyclic pentapeptide BQ123; its deficiency leads to pseudoxanthoma elasticum (83, 84). MRPs play crucial roles in drug disposition and excretion, impacting drug toxicity and drug interactions, and understanding their functions and regulation can help minimize drug toxicity, prevent unfavorable interactions, and overcome drug resistance (85).

Increased levels of MRPs and NRF2 activation are linked to various cancers, including lung, colorectal, breast, and prostate cancers (86). Overexpression of MRPs (such as MRP1, MRP2, and MRP3), and high NRF2 activity enhance drug efflux in these cancers, leading to chemoresistance and poor response to treatments by effectively removing chemotherapy agents from cancer cells (22, 82). This dual role of MRPs and NRF2 complicates cancer treatment due to increased resilience and continued cancer cell proliferation (22, 82).

### Glutathione S-transferase

GSTs are a family of Phase II detoxification enzymes that conjugate GSH to a variety of electrophilic compounds, both endogenous and exogenous (87). There are two main superfamilies of GSTs: membrane-bound microsomal and cytosolic. Microsomal GSTs, which form homo- and heterotrimers, are key in metabolizing leukotrienes and prostaglandins. Cytosolic GSTs, highly polymorphic, can be classified into six classes: α, μ, ω, π, θ, and ζ (87). The π and μ classes regulate cellular survival and death signals via interactions with JNK1 and ASK1, which are activated in response to cellular stress. GSTs are involved in developing resistance to chemotherapy agents through direct detoxification and inhibition of the MAP kinase pathway (87).

Continuous NRF2 overactivation exerts its transcriptional activities, upregulates GST (18). The GST family of enzymes detoxifies harmful compounds by conjugating them with GSH. This enhances cancer cells’ ability to detoxify ROS and other harmful compounds, leading to increased cancer cell proliferation, survival, and migration, thereby promoting tumor growth and metastasis (7). Furthermore, it increases cancer cells’ resistance to chemotherapy and radiation by enhancing their antioxidant and detoxifying capacities.

In summary, the GSH and the activity of various NRF2-regulated enzymes such as NQO1, HMOX1, SRXN1, TXNRD1, MRPs, and GST play crucial roles in cellular processes and maintaining cellular health.

## The role of NRF2 activation in different female cancers

### NRF2 activation in cervical cancer

**Figure 3:**
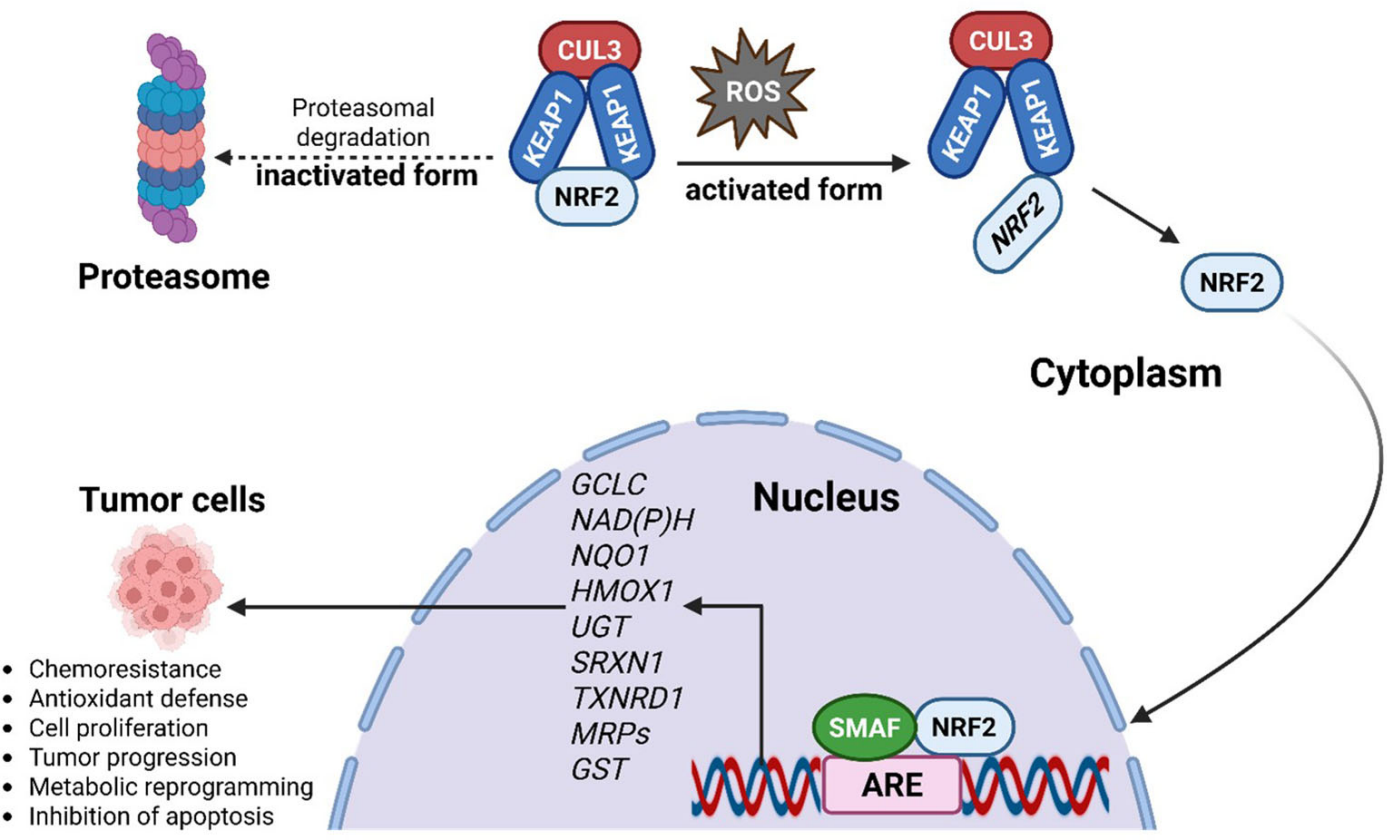
Distribution of female reproductive cancer cell types. Pink denotes gynecological malignancies; yellow marks breast cancer cells, which affect the reproductive system but are not considered gynecological.

Cervical cancer is one of the most prevalent cancers among women worldwide and originates in the cervical epithelial cells, which are divided into squamous and columnar epithelial cells (**Figure 3**, **Table 1**). These cells line the outer part (ectocervix) and the inner part (endocervix) of the cervix, the lower section of the uterus that connects to the vagina (92). The majority of cervical cancers are squamous cell carcinomas affecting the ectocervix. Adenocarcinoma, which affects the endocervix, is less common (93). The primary cause and significant risk factor for cervical cancer is infection with the human papillomavirus (HPV), which induces cellular changes that can lead to cancer development (94, 95).

**Table 1:** The location and function of cell types affected by female cancers.

Normal cell types	Functions	Location	Cancer cell types	References
Squamous epithelial cell 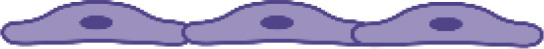	Protection from evaporation and external forces	Uterus Urethra Vulva Bladder Kidney Reproductive system	Squamous cell carcinoma	(88)
Endometrium 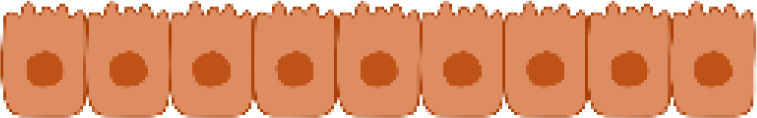	Cycle preparation Menstrual shedding Adhesion prevention	Uterus	Endometroid adenocarcinoma	(88, 89)
Germ cells 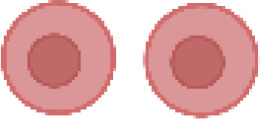	Create reproductive cells	Ovaries Testes	Germ cell carcinoma	(88)
Stroma cells 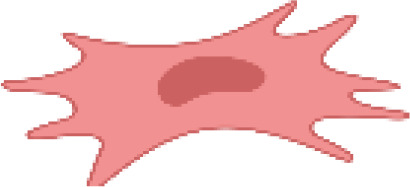	Structural support Immune regulation Tissue repair and regeneration	Ovaries Prostate	Stroma cell carcinoma	(88)
Basal cells 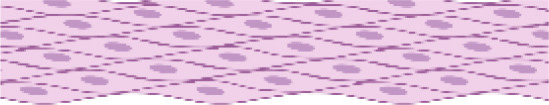	Cell regeneration Protection and regulation	Skin Airways	Basal cell carcinoma	(88)
Epithelial cells 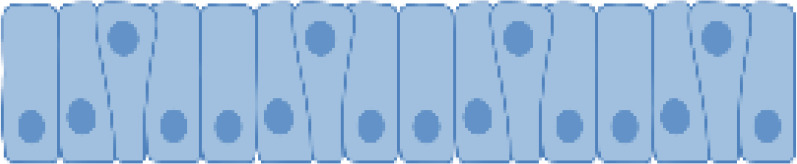	Secretion Absorption Protection Sensory reception	Nose Bronchi Uterus Fallopian tubes	Epithelial cell carcinoma	(88)
Mucin or goblet cells 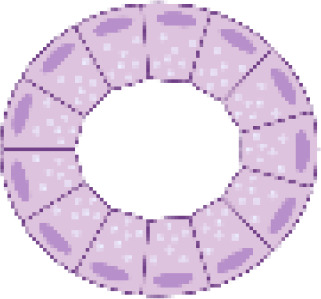	Lubrication Protection Mucociliary clearance Barrier Function	Respiratory tract Gastrointestinal tract Endometrium	Mucinous carcinoma	(88)
Serous epithelial cells 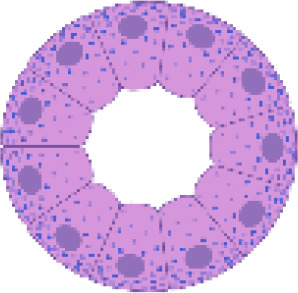	Lubricate organs Antimicrobial activity Antiviral activity Airway homeostasis	Uterus Mouth Pancreas Intestinal Crypts	Serous carcinoma	(88)
Lobules 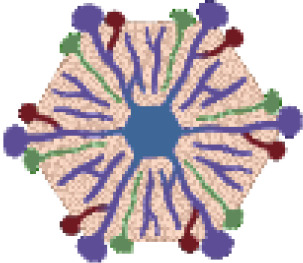	Sperm and testosterone production Milk production Liver function and breaking down fats	Liver Lungs Testicles Breast	Invasive lobular carcinomas	(90)
Ductal cells 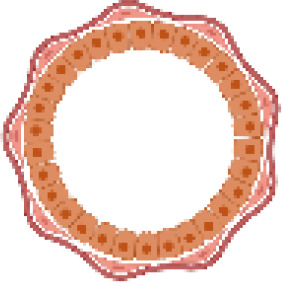	Milk transportation	Breast	Invasive ductal carcinomas	(91)

NRF2 activation plays a significant role in cervical cancer. In the early stages of cervical cancer development, NRF2 activation can prevent oxidative DNA damage and reduce the risk of mutations. NRF2-induced antioxidant genes, such as NQO1, HO-1, and GCLC, help mitigate oxidative stress, which is often elevated in cancer-prone tissues (14, 23). However, in advanced stages, persistent or aberrant activation of NRF2 can promote cancer cell survival, proliferation, and chemoresistance by enhancing antioxidant defenses (96). Studies have shown that cervical cancer tissues often exhibit increased nuclear NRF2 expression and decreased cytoplasmic KEAP1 expression. This imbalance leads to enhanced NRF2 activity, promoting cancer cell proliferation, inhibiting apoptosis, and increasing migration and invasion. Epigenetic changes, such as hypermethylation of the KEAP1 gene promoter, are also associated with increased NRF2 activity in cervical cancer (24, 96). Consequently, NRF2 may serve as a marker of poor prognosis and a potential therapeutic target in cervical cancer treatment (96).

There is considerable interplay between NRF2 activation and inflammatory pathways involving NF-κB and STAT3 (97). NF-κB, a key regulator of inflammation, can be activated by pro-inflammatory cytokines such as TNF-α, IL-6, and IL-1β (98). This activation typically leads to the production of ROS, which can, in turn, activate NRF2. In HPV-related cervical cancer, persistent infection may also induce chronic inflammation, further activating NF-κB and creating a pro-inflammatory environment (99). Consequently, NRF2 upregulates antioxidant and detoxifying genes, promoting cancer cell survival, proliferation, and resistance to therapy. STAT3, a transcription factor involved in cell proliferation, survival, and inflammation, can also be activated by cytokines like IL-6 and IL-1β. Once activated, STAT3 can induce the expression of NRF2, enhancing the cancer cells’ antioxidant and detoxifying capacity (18, 100–102). This interaction aids cancer cells in surviving oxidative stress and resisting therapy, contributing to tumor progression and chemoresistance. These pathways collectively create a more resilient and aggressive tumor microenvironment, posing challenges for effective cancer treatment. Understanding this crosstalk between NRF2, NF-κB, and STAT3 pathways can help in developing targeted therapies to disrupt these interactions and improve treatment outcomes for cervical cancer patients.

Targeting the NRF2 pathway in cervical cancer involves balancing its dual roles. In advanced cervical cancers with abnormal NRF2 activation, inhibitors like brusatol or ML385 can increase sensitivity to chemotherapy and radiation by reducing antioxidant defenses (14). Conversely, in early-stage cancer or pre-cancerous conditions, NRF2 activators such as sulforaphane may protect normal cells and prevent tumor initiation (14, 103). However, finding the right balance between activation and inhibition is challenging. Overactivation can promote tumor growth, while excessive inhibition may damage normal cells. Additionally, genetic and epigenetic variability among patients, resistance mechanisms, potential side effects, and the difficulties of translating preclinical findings to clinical applications further complicate the development of effective NRF2-targeted therapies. Addressing these challenges requires a nuanced approach, combining NRF2 inhibitors and activators with other therapeutic strategies to optimize treatment outcomes for cervical cancer patients.

### NRF2 activation in uterine or endometrial cancer

Endometrial cancer, originating in the inner lining of the uterus, is classified into Type I and Type II. The most common Type I cancer is endometrioid adenocarcinoma, which originates in glandular cells (**Figure 3**). Type II cancers, which are non-estrogen-dependent, include serous, mixed, squamous cell, uterine, and adenosquamous carcinomas (**Figure 3**; **Table 1**). Serous and clear cell carcinomas, although less common, are more aggressive and have high recurrence rates (104). Mixed carcinoma involves a combination of different cancer cells, while squamous cell carcinoma and uterine carcinosarcoma are rare types originating from different cell types. Adenosquamous carcinoma includes both glandular and squamous cells.

The role of NRF2 in endometrial cancer is shaped by its interactions with oxidative stress, the tumor microenvironment, and metabolic reprogramming (23). In the early stages, NRF2 activation protects normal endometrial cells from oxidative damage and genotoxic stress, reducing the risk of malignant transformation (20). However, in advanced endometrial cancer, mutations in the KEAP1-NRF2 pathway or epigenetic changes can lead to persistent NRF2 activation (105). These alterations increase the antioxidant capacity of cancer cells, enabling them to thrive under oxidative stress and resist chemotherapy and radiotherapy by mitigating treatment-induced oxidative damage (105).

NRF2 activation is particularly observed in Type II endometrial cancers, especially serous and clear cell carcinomas, and is associated with increased aggressiveness, poor prognosis, and chemoresistance (104, 106). For instance, a study found that 89% of endometrial serous carcinoma (ESC) cases exhibited positive NRF2 expression, whereas only 28% of endometrioid carcinoma cases showed similar levels (107). Recognizing NRF2’s function as a transcription factor with known target genes, Beinse et al. investigated the mRNA levels of three such genes (NQO1, GCLC, and AKR1C3). They discovered that ESC shows low NQO1 expression, which might be due to an interaction between NRF2 and TP53, potentially contributing to the cancer’s aggressiveness (108).

Cell line studies indicated that SPEC-2 cells (derived from ESC) had higher NRF2 levels and were more resistant to chemotherapy than Ishikawa cells (derived from endometrioid carcinoma) (14). Silencing NRF2 in SPEC-2 cells significantly increased their sensitivity to cisplatin and paclitaxel, highlighting the potential of targeting NRF2 to overcome chemoresistance.

Immunohistochemical analysis has shown elevated levels of NRF2 in Type II variants of endometrial cancer tissues, linked to increased cancer aggressiveness. Specifically, NRF2 is significantly overexpressed in ESC tissues compared to other endometrial tumors and benign tissues, indicating a direct correlation between NRF2 levels and cancer aggressiveness (107) High NRF2 levels in these cancers also confer increased resistance of SPEC-2 cells to chemotherapy drugs like cisplatin and paclitaxel (107).

Strategies like downregulating NRF2 through siRNA transfection or overexpressing KEAP1 have shown potential in making resistant cancer cells more responsive to treatment. This resistance was mitigated by transiently transfecting NRF2 siRNA or stably overexpressing KEAP1 in both *in vitro* and xenograft models (107). NRF2 promotes the expression of genes involved in detoxification and antioxidant defense, helping cancer cells survive despite treatment. Compounds such as brusatol and ML385 are being studied to reduce NRF2 activity in cancers with constant activation (109). These inhibitors may lower antioxidant defenses, making cancer cells more vulnerable to oxidative damage and increasing their sensitivity to chemotherapy and radiotherapy (110). In pre-cancerous conditions or early-stage cancer, activating NRF2 with natural compounds like sulforaphane and curcumin might help protect normal cells from oxidative stress and inflammation (111). Therefore, targeting NRF2 could potentially improve the effectiveness of chemotherapy in endometrial cancers.

### NRF2 activation in ovarian cancer

More than 95% of ovarian cancers are epithelial cell tumors (**Figure 3**; **Table 1**). These epithelial cells form the outer lining of the ovaries, which are the reproductive glands responsible for producing eggs and hormones like estrogen and progesterone (112). Ovarian cancer is especially dangerous because it is frequently diagnosed at a late stage (113). Genetic alterations are crucial in the development of ovarian cancer. While many ovarian cancers arise from random genetic mutations that build up over a person’s life, a significant portion (20-25%) is due to inherited genetic changes (23). For example, mutations in the BRCA1 and BRCA2 genes greatly elevate the risk of developing ovarian cancer (23).

Women with BRCA1 and BRCA2 mutations have a higher risk of developing breast and ovarian cancers, which tend to occur at younger ages (23, 114). The link between BRCA1 and BRCA2 mutations and NRF2 regulation lies in the increased oxidative stress caused by defective DNA repair mechanisms (23, 115). This oxidative stress can activate NRF2, promoting cancer cell survival and resistance to treatment (23, 115). Understanding the interplay between these genetic alterations and transcriptional regulation is crucial for developing targeted therapies for female cancers (23).

BRCA1 interacts with NRF2 to regulate antioxidant signaling by physically interacting with NRF2, promoting its stability and activation (116). NRF2 is a master regulator of antioxidant and cytoprotective genes that help both healthy and tumor cells cope with oxidative stress (117). The role of BRCA1 in regulating NRF2 activity has implications for the etiology and treatment of BRCA1-related cancers (116, 118).

NRF2 activation in ovarian cancer can have both positive and negative effects. Silencing NRF2 in ALDH1-enriched ovarian cancer cells significantly diminished their self-renewal capacity and stemness markers (119). This suggests that while NRF2 protects normal cells from oxidative damage, its overactivation in cancer cells may contribute to more aggressive tumor behavior. In early stages, NRF2 protects ovarian cells from oxidative stress and DNA damage due to ovulation-associated tissue damage and repair, potentially preventing cancer (43, 120). In advanced ovarian cancer, dysregulation of the Keap1-Nrf2 pathway leads to persistent NRF2 activation, promoting chemoresistance and immune evasion (120).

NRF2 and NQO1 were highly expressed in ovarian cancer and precancerous tissues compared to normal tissues, showing a positive correlation across lesion types (120, 121). NRF2 was present in both the nucleus and cytoplasm of ovarian cells, with its levels increasing as the cancer advanced (121).

Targeting NRF2 in ovarian cancer is challenging, but inhibitors like ML385 and brusatol are being investigated for cancers with abnormal NRF2 activation (122). These inhibitors enhance the sensitivity of cancer cells to chemotherapy by reducing their antioxidant defenses and increasing oxidative stress, making them more susceptible to apoptosis. Combining NRF2 inhibitors with conventional therapies, such as cisplatin and carboplatin, has shown promise in preclinical studies (122). It was suggested that targeting glutamate-cysteine ligase catalytic subunit (GCLC) could be an effective therapeutic strategy for ARID1A-deficient ovarian cancers (43). They identified GCLC as a synthetic lethal target in ARID1A-deficient ovarian and gastric cancers using CRISPR knockout technology. ARID1A mutations increase the sensitivity of these cancer cells to GCLC depletion, indicating that targeting GCLC could selectively attack cancer cells while sparing normal cells, thereby reducing the risk of excessive ROS buildup and DNA damage (43). Other genes that may be implicated in ovarian cancers include BRCA1 interacting protein C-terminal helicase 1 (BRIP1), MutL homolog 1 (MLH1), MutS homolog 2 (MSH2), MutS homolog 6 (MSH6), postmeiotic segregation increased 2 (PMS2), epithelial cell adhesion molecule (EPCAM), partner and localizer of BRCA2 (PALB2), RAD51 paralog C (RAD51C), and RAD51 paralog D (RAD51D) (123, 124).

### NRF2 activation in vaginal cancer

Vaginal cancer is an uncommon type of cancer that affects the cells lining the vagina, the muscular canal connecting the cervix to the external genitalia (125, 126). It represents a small fraction (1-2%) of gynecological cancers, with the majority of cases being squamous cell carcinomas (**Figure 3**; **Table 1**), and is most frequently diagnosed in older women (127). Many instances of vaginal cancer are associated with DNA changes caused by HPV. For example, DNA damage response (DDR) mutations, which hinder the cell’s ability to repair DNA damage, have been identified through genomic sequencing. These mutations promote cancer growth and make cancer cells more dependent on specific repair pathways, such as homologous recombination repair (HRR), nucleotide excision repair (NER), and mismatch repair (MMR). The same mutations may also rely on NRF2 activation for DNA repair and survival (128).

Although the role of NRF2 activation in vaginal cancer remains largely unexplored, other transcription factors have been implicated in its pathogenesis. Studies have shown that P63, a transcription factor commonly associated with squamous epithelial cells, is overexpressed in vaginal tumors, indicating its involvement in vaginal squamous cell carcinoma in mouse models (129). Similarly, P53, an important transcription factor responsible for maintaining genome integrity by preventing the replication of damaged DNA, is frequently mutated in malignant vaginal tumors, highlighting its significance in tumor suppression and cancer progression (84, 130). Mutations in the *P53* gene are often found in malignant vaginal tumors (131).

More insights can be drawn from cancers with similar histologic forms. Vaginal cancer predominantly presents as squamous cell carcinoma (SCC), which makes up approximately 90% of all cases (126). In other SCCs, such as those of the head, neck, and oesophagus, NRF2 is often constitutively activated, contributing to tumor progression and therapy resistance (132). For example, Hamad and colleagues demonstrated that NRF2 activation, in the absence of tumor suppressors *p15* and *p16*, was essential for the development and progression of oral SCC in mouse models (133). Additionally, dysregulated NRF2 has been implicated in resistance to chemoradiotherapy in esophageal SCC (134, 135). Specifically, NRF2 was shown to enhance radiation resistance in esophageal SCC by upregulating the CaMKIIα gene, which promotes autophagy and facilitates cancer cell survival (136). Given these findings, it is plausible that NRF2 may play a similar role in vaginal squamous cell carcinoma by promoting tumor development and therapy resistance through similar molecular pathways.

Interestingly, research has revealed a potential link between NRF2 and P63, specifically its isoform Np63, which interacts with NRF2 to maintain cellular homeostasis and prevent tumor formation (137). Another study further demonstrated that NRF2 and P63 interact to promote keratinocyte proliferation in the epidermis. The presence of promoters and enhancers co-activated by both transcription factors suggests a coordinated role in epithelial renewal, which could extend to tumor development and progression in vaginal tissues (138). These findings suggest that NRF2, through its interactions with key transcription factors such as P63 and P53, may play an indirect role in vaginal cancer. Targeting NRF2 activity with inhibitors like brusatol and ML385 could be beneficial (139). This may enhance chemotherapy and radiation effectiveness by reducing antioxidant defenses and increasing oxidative stress in tumor cells (14). Combining these inhibitors with standard treatments could also improve outcomes by overcoming resistance mechanisms (14).

### NRF2 activation in vulvar cancer

Most vulvar cancers are squamous cell carcinomas, originating in the thin, flat cells lining the vulvar surface (**Figure 3**; **Table 1**). These rare cancers develop in the external female genitalia, including the labia, clitoris, and vaginal opening (140). They primarily affect older women, though they can occur at any age (140). Vulvar cancer is relatively uncommon compared to breast and certain gynecological cancers, making early detection crucial for successful treatment (141). HPV infection is a significant risk factor, as it can cause genetic mutations that lead to cancerous changes in vulvar cells. Mutations in genes like *TP53* and DNA repair genes can disrupt cell cycle regulation and impair the cell’s ability to repair DNA damage, resulting in genetic errors (142, 143). Additionally, epigenetic changes can contribute by silencing tumor suppressor genes or activating oncogenes, facilitating the progression of vulvar cancer (144).

Vulvar cancer, though rare, is aggressive with distinct molecular features implicating NRF2 activation in its development, progression, and treatment resistance (15). For instance, disruptions in the KEAP1-NRF2 regulatory axis are a primary mechanism of NRF2 dysregulation (145). KEAP1 mutations, frequently observed in vulvar squamous cell carcinoma, are associated with poor prognostic outcomes (146–148).

NRF2’s role in metabolic reprogramming contributes to vulvar cancer pathophysiology by driving gene expression in the pentose phosphate pathway and glutathione biosynthesis, providing cancer cells with metabolic flexibility for rapid proliferation (23). These shifts enable tumors to adapt to hypoxic conditions, presenting challenges in targeting NRF2-driven tumors (149).

NRF2 regulates cellular redox balance and interacts with pathways such as phosphoinositide 3-kinase/protein kinase B (PI3K/AKT), Notch, hypoxia-inducible factors (HIFs), nuclear factor kappa-light-chain-enhancer of activated B cells (NF-κB), myelocytomatosis oncogene (MYC), and forkhead box M1 (FOXM1), promoting tumor progression by enhancing EMT, angiogenesis, and immune evasion (18, 150–152). Elevated NRF2 levels are often associated with fewer immune cells in tumors, aiding cancer survival and spread (153). Although these interactions have not been specifically reported in vulvar cancer, they likely contribute to its aggressive nature and treatment resistance, warranting further investigation.

While targeting NRF2 presents challenges, it offers a promising avenue for improving treatment outcomes. Preclinical studies have explored small-molecule inhibitors that target NRF2 or its downstream effectors and agents that restore KEAP1 function to suppress NRF2 activity. These strategies show potential in reversing chemoresistance and sensitizing tumors to radiation (154). Combining NRF2 inhibitors with immune checkpoint blockade therapies may enhance anti-tumor immunity. Further research is needed to understand NRF2’s complex roles in vulvar cancer and identify biomarkers for personalized treatment.

### NRF2 activation in breast cancer

Most breast cancers are invasive ductal carcinomas (**Figure 3**; **Table 1**), which are common and deadly, ranking fifth in cancer-related mortality worldwide (155). Breast cancer originates in the breast tissue, specifically in the ducts or lobules. Although not classified as a gynecological cancer, breast cancer significantly impacts female reproductive health. It is classified based on estrogen receptor (ER), progesterone receptor (PR), and human epidermal growth factor receptor 2 (HER2) status, which guide treatment (3, 4, 114). Risk factors include gender, age, hormonal status, genetic factors, and ionizing radiation (156). Prolonged estrogen exposure increases risk, especially with early menstruation and late menopause (156). Mutations in BRCA1 and BRCA2 genes, crucial for DNA repair, lead to breast cancer and genomic instability (4, 114, 116, 156).

NRF2 activation in breast cancer exhibits a dual role (24). In normal cells, NRF2 upregulates antioxidant and detoxifying genes, limiting oxidative DNA damage and preventing carcinogenesis, but in malignant cells it can promote survival, proliferation, and resistance to chemotherapy and radiotherapy (15). Somatic mutations, such as KEAP1 C23Y, impair NRF2 repression and drive its stabilization and persistent antioxidant signaling (157, 158). NRF2 also cross-talks with Notch pathways, contributing to invasion and therapy resistance (14, 65).

Importantly, NRF2 activity in breast cancer is modulated by sex-specific and hormonal influences. In ER-negative tumors, high NRF2 expression suppresses CXCL13 and limits proliferation, whereas in ER-positive tumors, low NRF2 levels increase CXCL13/CXCR5 expression, promoting invasion and metastasis (65). In BRCA1-deficient cells, ROS accumulation reflects a compromised NRF2 response, but reactivation of NRF2 restores survival. Estrogen protects BRCA1-deficient mammary cells from oxidative stress-induced death by activating NRF2 via the PI3K–AKT pathway (158). Consistently, NRF2–Keap1 signaling is more active in ER-, PR-, and HER2-positive tumors compared to TNBC (159).

Beyond breast cancer, female-specific vulnerabilities emerge in ovarian cancers associated with BRCA mutations, where disrupted NRF2-BRCA1 interactions compromise redox defenses and reveal unique therapeutic windows, reviewed in Li et al (14). These observations highlight that NRF2 regulation is not only context- but also sex-dependent, shaped by hormonal environment and receptor status. As reviewed by Adinolfi et al (17), NRF2 protects against tumor initiation but supports progression once malignancy is established. Thus, while NRF2 is a promising therapeutic target, interventions must be carefully tailored to the sex-specific and hormonal context to avoid unintended tumor-promoting effects (17).

NRF2 activation in breast cancer enhances the expression of Rho and its downstream proteins like Focal adhesion kinase 1 (FAK) and Modulator of volume-regulated anion channel current 1 (MLC), while suppressing estrogen-related receptor α (ERR1) (23). NRF2 can interact with BRCA1, enhancing its stability. In the absence of BRCA1, estrogen restores NRF2 activation, reducing ROS levels and protecting mammary gland cells *in vitro* (23). Continuous NRF2 signaling in cancer promotes malignant progression, therapy resistance, and poor clinical outcomes. Elevated NRF2 levels in breast cancer patients are linked to lower overall and disease-free survival. While NRF2 supports oncogenic signaling and cancer progression, it also plays a chemopreventive role in normal cells by suppressing ROS-induced DNA damage and carcinogenesis, highlighting its dual role (160, 161).

## Therapeutic challenges and avenues

Emerging evidence indicates that the role of NRF2 in cancer extends far beyond redox regulation, presenting significant therapeutic challenges. Constitutive activation of NRF2, often resulting from KEAP1 mutations, facilitates immune evasion by suppressing interferon signaling, altering cytokine expression, and downregulating antigen-presentation pathways (13, 14). These changes foster immune-cold tumor microenvironments, particularly in cancers with limited T-cell infiltration. In parallel, NRF2 promotes metabolic reprogramming through enhanced glutaminolysis and pentose phosphate pathway activity, enabling tumor cells to withstand oxidative and metabolic stress (14). Its transcriptional regulation of drug efflux transporters and detoxification enzymes further contributes to broad-spectrum chemoresistance (14, 41).

**Figure 4:**
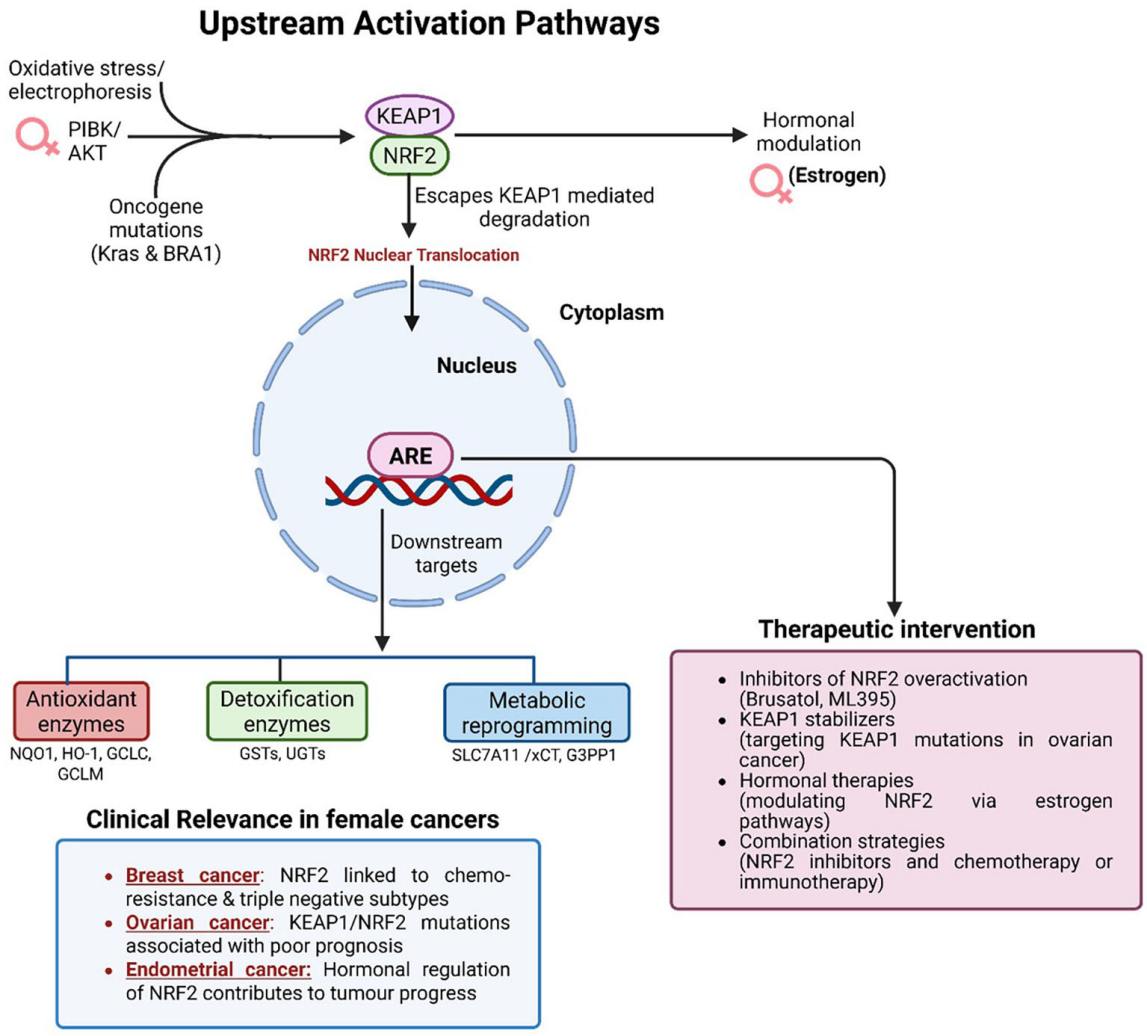
Upstream activation pathways and therapeutic targeting of NRF2 in female cancers. Oxidative stress, oncogenic mutations, and PI3K/AKT signaling disrupt KEAP1-mediated degradation, allowing NRF2 nuclear translocation and activation. Estrogen further modulates NRF2 activity in hormone-responsive cancers. In the nucleus, NRF2 drives expression of antioxidant, detoxification, and metabolic genes that promote chemoresistance, immune evasion, and tumor progression. Aberrant NRF2 activation is particularly relevant in triple-negative breast cancer, platinum-resistant ovarian cancer, and endometrial carcinoma. Therapeutic strategies include NRF2 inhibitors (Brusatol, ML385), KEAP1 stabilizers, hormonal therapies, and combination regimens with chemotherapy or immunotherapy.

In female-specific malignancies such as TNBC, platinum-resistant ovarian cancer and type II endometrial carcinoma, sustained NRF2 activation is consistently associated with poor outcomes, as illustrated in **Figure 4** (38, 39). This effect is driven by upregulation of antioxidant genes (e.g., HO-1, NQO1, GCLC), which neutralize chemotherapy-induced ROS and suppress apoptotic signaling (14). These context-dependent effects complicate therapeutic targeting: while NRF2 inhibition may restore sensitivity to cytotoxic agents, it simultaneously risks compromising protective functions in normal tissues.

Therapeutic strategies therefore require careful balancing of NRF2’s tumor-promoting versus cytoprotective roles. Selective modulation, ideally within genetically defined subgroups, represents a promising approach. Small-molecule inhibitors such as Brusatol (though its effects extend beyond NRF2, including global inhibition of protein synthesis) and ML385 have demonstrated efficacy in preclinical studies by suppressing NRF2-driven transcription and re-sensitizing tumors to chemotherapy and radiotherapy (**Figure 4**) (14, 17). Alternative strategies involve stabilizing KEAP1 or correcting KEAP1 loss-of-function mutations to re-establish upstream regulatory control (38).

Given the hormonal regulation of NRF2 in estrogen-responsive cancers, rational drug combinations are gaining attention. Integration of NRF2 inhibitors with endocrine therapies or immune checkpoint blockade may yield synergistic effects. Preclinical studies indicate that NRF2 inhibition enhances responsiveness to PD-1/PD-L1 inhibitors, particularly in poorly immunogenic tumors (15). These insights underscore the need for future clinical trials that incorporate NRF2 status as a predictive biomarker and test dual-modality regimens in stratified cohorts.

Synthetic lethality approaches further expand the therapeutic landscape. NRF2-overexpressing tumors display vulnerabilities to inhibitors of parallel survival pathways such as glutaminase and thioredoxin reductase (36, 40). Targeting these metabolic dependencies offers a precision-medicine strategy, particularly in genomically unstable or metabolically stressed tumors (37).

The expanding pipeline of NRF2 modulators highlights both opportunity and complexity. While small-molecule inhibitors like Brusatol and in **Table 2** ML385 remain leading candidates for NRF2-addicted tumors, natural compounds such as sulforaphane and curcumin are under investigation for chemoprevention (14).

**Table 2:** Therapeutic strategies targeting NRF2: inhibition versus activation.

Strategy	Context/rationale	Examples	Therapeutic goal	Status/limitations	References
NRF2 Inhibition	Constitutive NRF2 drives resistance and immune evasion.	Brusatol; ML385; KEAP1 stabilizers	Resensitize tumors to chemo, radio, immunotherapy	Preclinical; limited clinical data; specificity concerns	(14, 17)
Synthetic Lethality	NRF2-addicted tumors rely on parallel survival pathways.	Glutaminase and thioredoxin reductase inhibitors	Exploit metabolic vulnerabilities	Precision potential; mostly preclinical	(14)
NRF2 Activation (Chemoprevention)	In high-risk/premalignant settings, NRF2 boosts detox and DNA protection.	Sulforaphane; curcumin; bardoxolone methyl (NCT01826487)	Prevent carcinogenesis; reduce oxidative/carcinogen stress	Feasible; safety/toxicity issues	(17)
Combination Approaches	Dual targeting may enhance endocrine or immune therapies.	NRF2 inhibitors + endocrine or PD-1/PD-L1 blockade	Improve response in stratified subgroups	Preclinical support; needs clinical validation	(15)

Conversely, systemic NRF2 activators (e.g., bardoxolone methyl, NCT01826487 in **Table 2**) illustrate translational interest but also raise safety concerns related to off-target toxicity (17). Importantly, this underscores the therapeutic paradox of NRF2 modulation: in established tumors, NRF2 inhibition may overcome resistance and restore sensitivity to therapy, whereas in premalignant or high-risk contexts, NRF2 activation may serve as a chemopreventive strategy by enhancing cellular defenses against carcinogens. The central challenge remains to achieve tumor-specific NRF2 inhibition without undermining its protective role in healthy tissues, and to harness NRF2 activation for prevention without inadvertently fueling tumor progression.

## Positron emission tomography and radiotracers: potential in imaging NRF2

### Current imaging modalities of female cancers and limitations

Positron emission tomography (PET) is a highly effective molecular imaging technique that enables the visualization and measurement of biological processes in living subjects (162). This method relies on radiotracers, which are molecules labelled with positron-emitting isotopes, allowing them to be detected and tracked within the body (163). The development of radiotracers targeting specific molecular pathways involved in cancer has significantly advanced early detection, precise staging, prediction of treatment responses, and monitoring of therapeutic effectiveness (164, 165).

**Figure 5:**
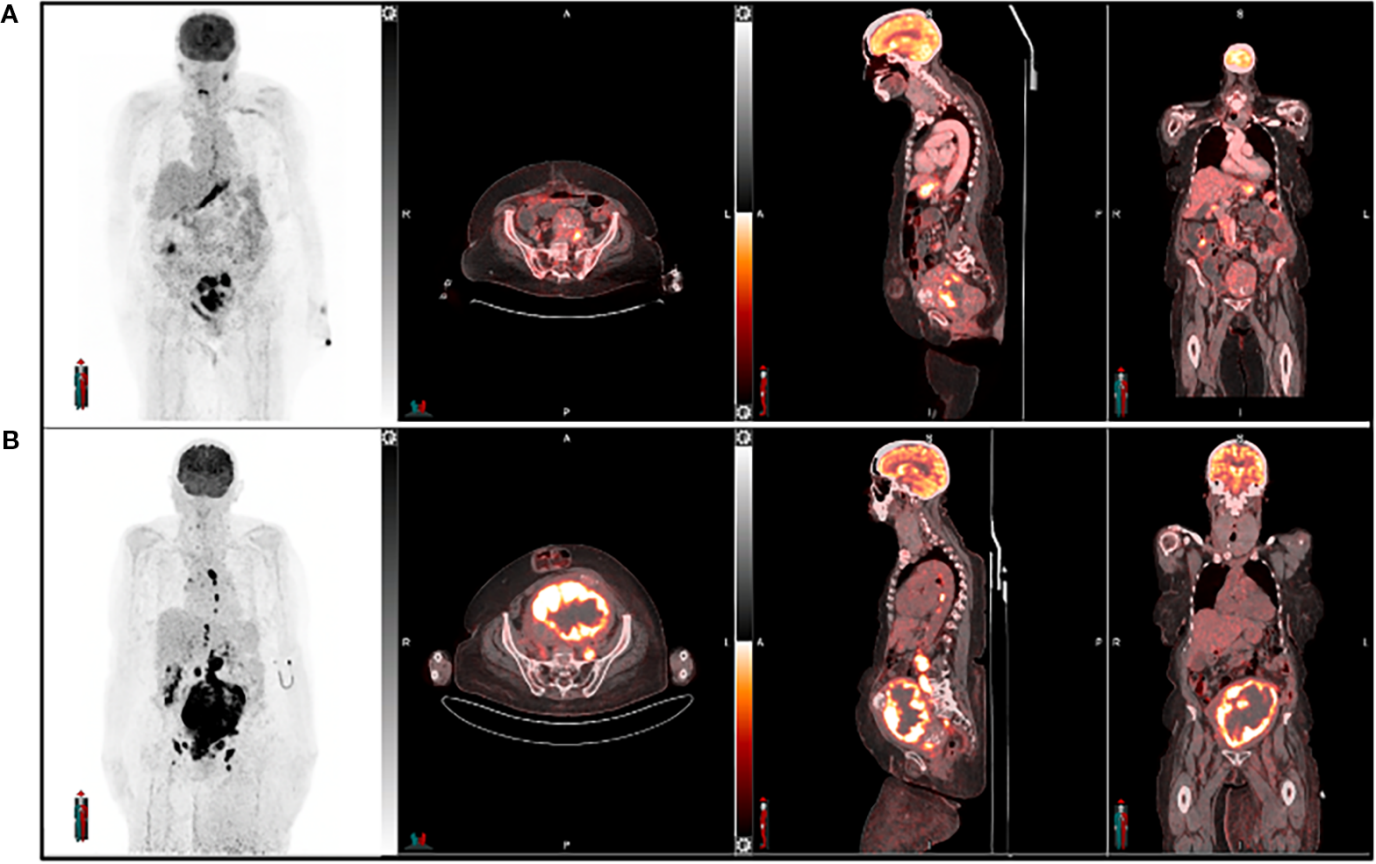
A 64-year-old female with endometrial cancer underwent [^18^F] FDG PET imaging for both diagnosis and management. The pre-treatment scan **(A)** was used for initial assessment, while the post-treatment scan **(B)** evaluated treatment response six weeks after therapy completion. Follow-up imaging revealed an increase in the size of the primary tumor and nodal metastatic lesions, along with heightened tracer uptake, suggesting disease progression or treatment failure.

^18^F-Fluorodeoxyglucose (^18^F-FDG) PET is a well-established imaging that plays a vital role in diagnosing, staging, treatment planning, and detecting recurrences in female cancers, particularly gynecological malignancies. When combined with computed tomography (FDG-PET/CT), it identifies increased glucose metabolism, a key characteristic of many cancers, allowing for precise tumor visualization and response assessment (**Figure 5**) (166, 167). FDG-PET/CT improves staging accuracy by detecting lymph node involvement and distant metastases, which are crucial for effective treatment planning (165, 168). Furthermore, it demonstrates high sensitivity in identifying recurrent disease, especially in patients with elevated tumor markers but no visible lesions on conventional imaging (165, 169).

Despite its benefits, FDG-PET/CT has several limitations. Certain low-grade tumors, such as mucinous ovarian cancers, exhibit low FDG uptake, leading to false-negative results (170). Additionally, metabolic changes induced by chemotherapy or radiotherapy can further influence FDG uptake, making treatment response assessment more challenging. Inflammatory conditions, including post-surgical changes, endometriosis, and pelvic inflammatory disease, can also cause false positives, complicating image interpretation (171–173). Furthermore, FDG-PET/CT has difficulty distinguishing viable tumors from inflammatory processes, assessing therapy resistance (including NRF2 status), and evaluating non-FDG-avid tumors (167, 174, 175). Moreover, ^18^F-FDG does not provide detailed insights into the molecular mechanisms driving tumor aggressiveness or treatment response. With the increasing shift toward personalized medicine and targeted cancer therapies, there is a growing demand for more specific PET tracers. These tracers could directly target receptors, signaling pathways, and molecular markers (e.g., ^18^F-FSPG) associated with tumor progression, metastasis, and therapy resistance. By offering a more precise and dynamic understanding of tumor biology, such advancements would enhance treatment monitoring, improve outcome prediction, and optimize therapy selection.

### Potential imaging modalities through NRF2

Given NRF2’s pivotal role in cancer progression and resistance to various therapies, including chemotherapy, radiotherapy, immunotherapy, and KRAS G12C inhibitors, there is increasing interest in developing PET tracers to visualize NRF2 activation *in vivo* (176, 177). Although PET imaging of NRF2 is still in its early stages, several promising strategies have emerged, which can be broadly categorized into direct and indirect approaches. Direct strategies focus on imaging the NRF2 protein itself, while indirect methods target downstream effectors or metabolic consequences of NRF2 activation.

Direct imaging of NRF2 has proven challenging. One approach involves developing radiolabeled small-molecule inhibitors of NRF2. Several NRF2 inhibitors, such as brusatol and trigonelline, have been identified (174, 178), but creating radiolabeled versions with suitable properties for PET imaging has been difficult. Another direct strategy is the development of radiolabeled antibodies or antibody fragments targeting NRF2. While this approach offers high specificity, it faces significant challenges, including NRF2’s intracellular localization and the need for tracers to effectively cross the cell membrane (179).

Indirect imaging approaches provide valuable insights into tumor biology, especially when direct visualization of specific molecular targets is challenging. Tracers such as ^18^F-FDG and ^68^Ga-FAPI serve as surrogates for more specific biomarkers, offering indirect assessments of key tumor processes. For instance, in hypoxic tumor regions, where oxygen deprivation drives aggressive cancer behavior, ^18^F-FDG uptake increases due to enhanced glycolytic activity, making it a useful surrogate for hypoxia (180). Similarly, ^68^Ga-FAPI, which targets fibroblast activation protein (FAP), helps visualize tumor-associated stroma and its role in cancer progression, including hypoxia-induced changes in the tumor microenvironment (165). These surrogate markers are especially valuable when direct imaging of specific molecular pathways is challenging or not feasible. They play a crucial role in the advancing field of theragnostics, improving tumor characterization and optimizing treatment planning.

**Figure 6:**
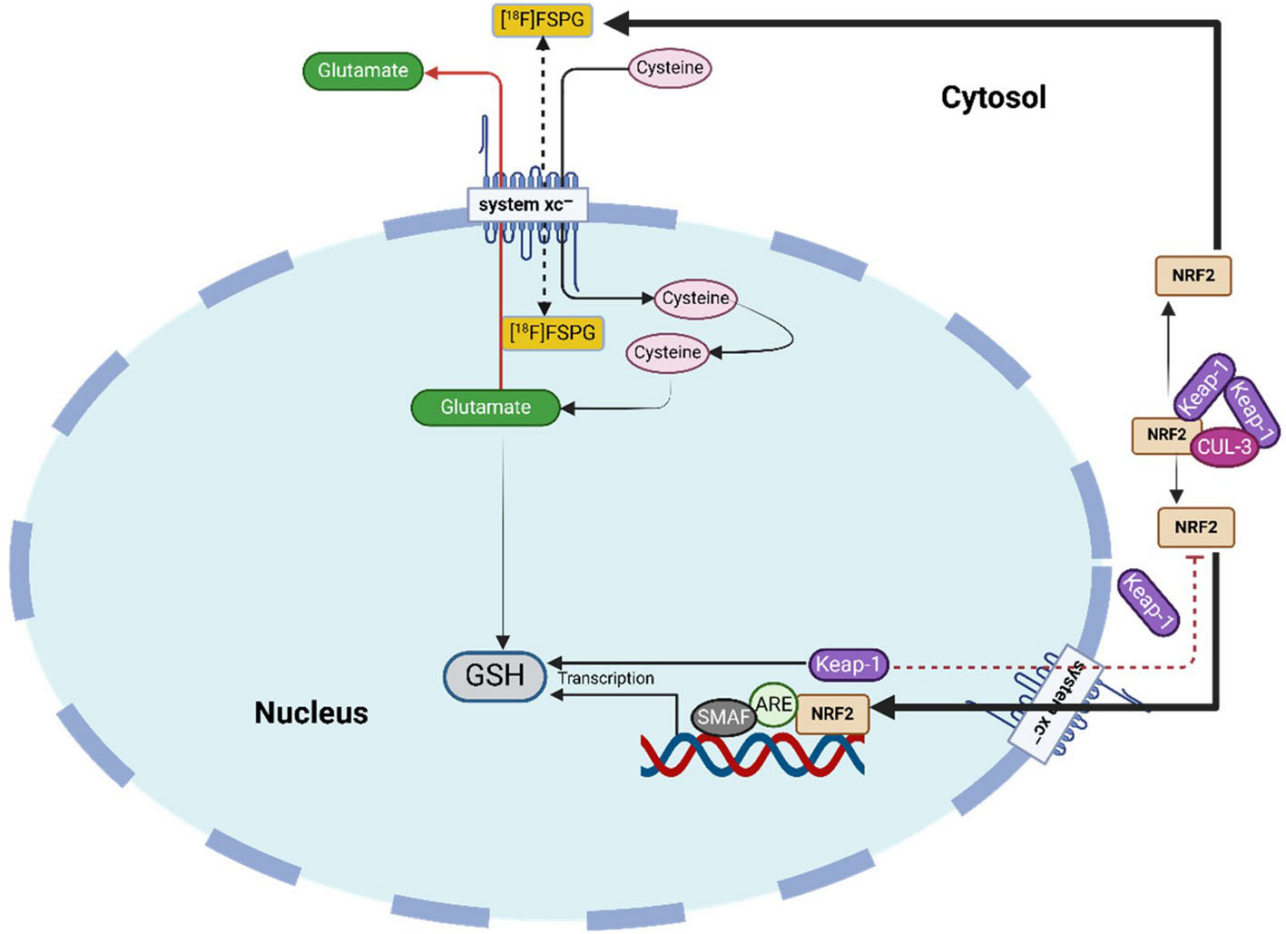
System xc^-^ tracer uptake as an indirect marker of NRF2 activity and therapy response. NRF2 enhances system xc^-^-mediated cystine import for GSH synthesis; oxidative stress from treatment can reduce uptake, while resistant tumors maintain high tracer retention.

An indirect strategy for imaging NRF2 activity involves targeting the cystine/glutamate antiporter system xc^-^, which is upregulated by NRF2 activation (181). Under oxidative stress or antioxidant depletion, tumor cells enhance antioxidant production, such as GSH, to maintain redox balance, as illustrated in **Figure 6** (117). This adaptive response is regulated by NRF2 and its negative regulator KEAP1, which influence transmembrane transporters like system xc^-^ (182). System xc^-^ facilitates cystine uptake in exchange for glutamate, with cystine serving as a precursor for GSH synthesis (183). Several PET tracers have been developed to assess system xc^-^ activity, including 4-^18^F-fluoroglutamate (^18^F-FGlu) and (4S)-4-(3-^18^F-fluoropropyl)-L-glutamate (^18^F-FSPG) (184, 185). Additional tracers capable of imaging oxidative stress include [^18^F]5-fluoro aminosuberic acid (FASu), [¹¹C]ascorbic acid ([¹¹C]VitC), and [¹¹C]dehydroascorbic acid ([¹¹C]DHA) (186, 187). FASu, which targets system xc^-^, has demonstrated potential for imaging tumor redox status in breast cancer (188). Meanwhile, [¹¹C]VitC and [¹¹C]DHA track ROS and vitamin C uptake in NRF2-driven tumors, where accumulation is notably increased (189, 190).

^18^F-FSPG has demonstrated potential in both preclinical and clinical studies for imaging system xc^-^ activity across various cancers, including lung, breast, ovarian, and head and neck cancers (191, 192). In NSCLC, ^18^F-FSPG uptake is associated with tumor aggressiveness and poor prognosis (174). Approximately one-third of NSCLC patients harbor NRF2/KEAP1 mutations, which contribute to poorer responses to standard therapies. The NRF2-regulated system xc^-^ transporter supports GSH production, providing cancer cells with a survival advantage. ^18^F-FSPG PET enables the detection of NRF2 activation and related metabolic alterations, offering a promising tool for targeted therapeutic strategies (174). Tumors with elevated NRF2 activity exhibit increased xCT expression, higher GSH levels, and lower ROS accumulation. Targeting xCT with drugs such as HM30-tesirine has shown efficacy in overcoming therapy resistance in NRF2-driven NSCLC tumors (174). Non-invasive assessment of NRF2 activation via PET imaging could improve predictions of therapy resistance and inform the development of precision medicine approaches for resistant cancers. Furthermore, ^18^F-FSPG PET has been utilized to monitor treatment response to therapies designed to inhibit system xc^-^ activity (112).

## Conclusion and future perspectives

NRF2 is a paradoxical regulator in female cancers: while transient activation safeguards normal tissues against oxidative stress and carcinogen-induced damage, persistent hyperactivation supports tumor growth, immune evasion, and therapy resistance. This duality underscores the central translational challenge, defining when NRF2 activation should be preserved for chemoprevention and tissue protection, and when suppression is required to overcome tumor aggressiveness.

Pharmacological modulators highlight both the promise and complexity of NRF2-directed strategies. Inhibitors such as brusatol and ML385 can re-sensitize NRF2-addicted tumors, while natural activators like sulforaphane and curcumin hold chemopreventive potential. However, safety concerns from systemic activators (e.g., bardoxolone methyl) emphasize the need for selective and context-specific modulation that balances tumor suppression with preservation of physiological defenses. Hormonal regulation adds an additional layer, with estrogen-linked NRF2 activation in BRCA1-deficient contexts demonstrating the necessity of sex- and genotype-informed therapeutic design.

Emerging tools, particularly PET tracers such as ^18^F-FSPG, offer new opportunities to non-invasively monitor NRF2 activation, identify resistant tumors, and guide patient stratification. The integration of NRF2 imaging with molecular profiling could enable earlier detection, dynamic response monitoring, and refined precision therapies. Future advances will depend on multidisciplinary efforts to achieve context-dependent NRF2 modulation, ensuring that its protective benefits are harnessed while its tumor-promoting functions are contained, ultimately improving outcomes for women with breast, ovarian, and endometrial cancers.
